# Supplementary material for: Strain-Induced Alignment in Collagen Gels
Source: PLoS One. 2009 Jun 16;4(6):e5902. doi: 10.1371/journal.pone.0005902 (PMC2691583; doi:10.1371/journal.pone.0005902)
Supplement: Methods S1 — Validation of fiber orientation algorithm (0.02 MB DOC) [file pone.0005902.s001.doc]

## Validation of fiber orientation algorithm

The image processing method described in the manuscript to extract local collagen fibril orientation from confocal images has been validated on computer-made random networks of sticks on a 2D plane. A large number of sticks of a given length are drawn at random (position and orientation) on an image (see Figure S1A) to simulate the appearance of the experimental images of collagen network. In addition, image blur and Gaussian noise have been introduced (Figure S1B). These images are then submitted to the analysis described in the paper. Figure S1C shows the local fiber orientation at every location where fiber could be non-ambiguously detected.

We then compare the true histogram of stick orientations, which is calculated while the original image is created, with the angular distribution calculated by the image analysis method (Figure S2A). To determine whether the two histograms - the true one and the measured one - are from the same probability distribution, we use the Kolmogorov-Smirnov test (Lehmann and Romano, 2005), which is based on the comparison of the two cumulative probability distributions (see Figure S2B). In all validation tests conducted on simulated images of densities comparable to our confocal data, we find that the Kolmogorov-Smirnov test does not allow us to reject the null hypothesis that the two histograms are from the same probability distribution function (*p*-value >0.05). In most, but not all cases, the *p*-value is even greater than 0.2; the example illustrated in Figure S2 and Figure S2 has *p*>0.2.

**References**

Lehmann, E. L., and J. P. Romano, 2005. Testing Statistical Hypotheses. Springer.
