# Supplementary figures and images for: Strain-Induced Alignment in Collagen Gels
Source: PLoS One. 2009 Jun 16;4(6):e5902. doi: 10.1371/journal.pone.0005902 (PMC2691583; doi:10.1371/journal.pone.0005902)

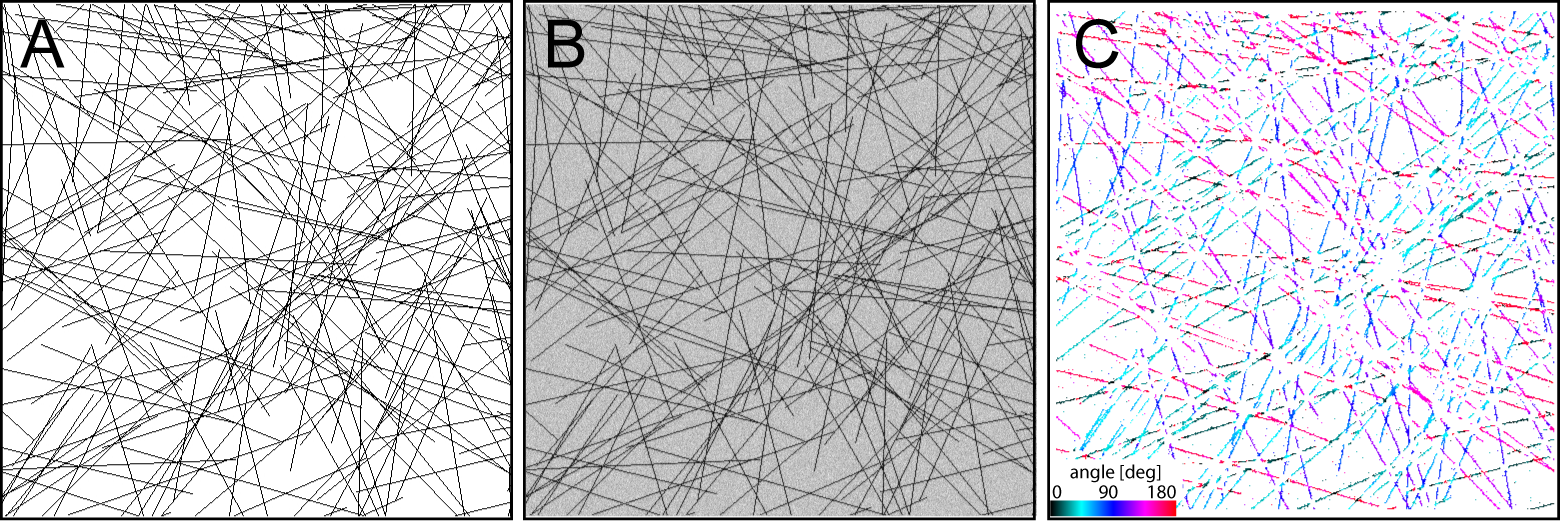

Supplement: Figure S1 — (A) Example of a simulated random network of sticks in two dimensions. (B) Same example, after blurring and added Gaussian noise. (C) Color-coded local stick orientation, as determined by our image processing algorithm. Angle color scale at bottom. (0.80 MB TIF) [file pone.0005902.s002.tif]

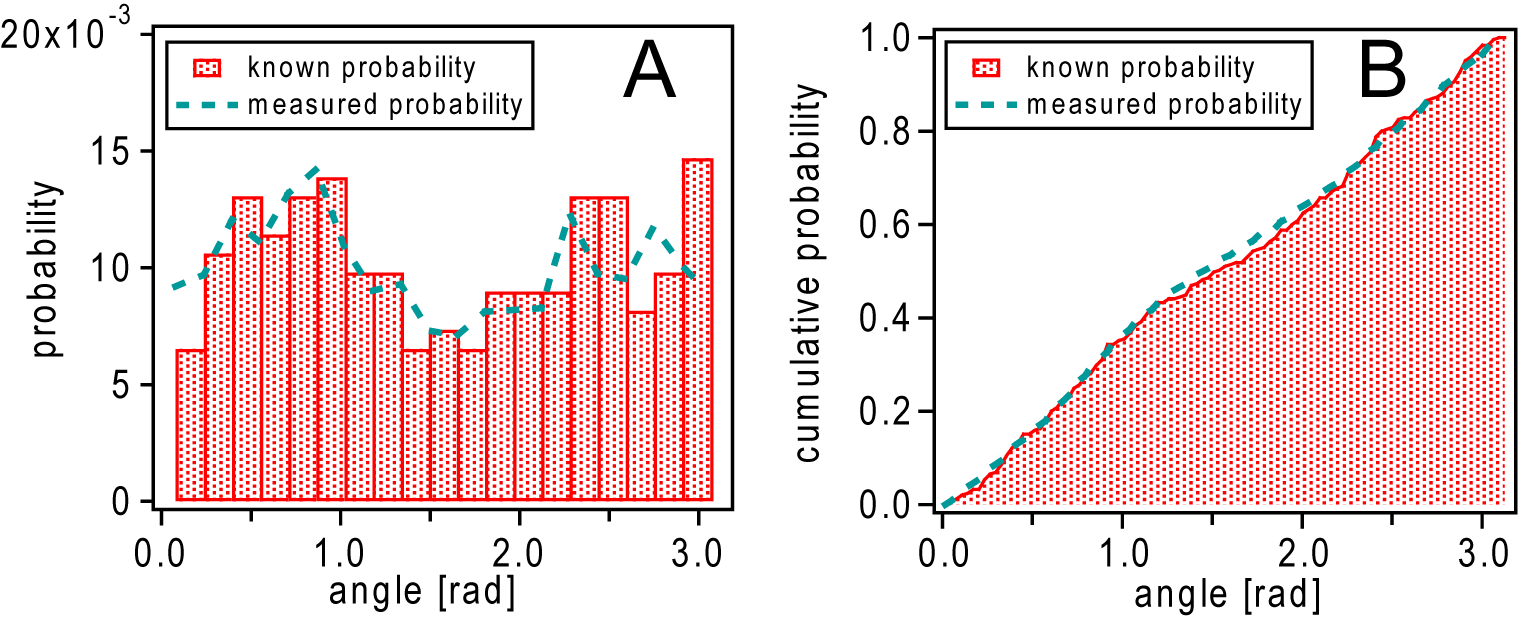

Supplement: Figure S2 — Known and calculated (A) probability density functions and (B) cumulative distribution functions of the orientation of individual sticks, based on image shown in Figure S1A. (0.23 MB TIF) [file pone.0005902.s003.tif]
